# Estimation of above-ground carbon stock of *Dendrocalamus giganteus* using ANUSPLIN-interpolated GEDI and ICESat-2/ATLAS parameters

**DOI:** 10.3389/fpls.2025.1676195

**Published:** 2025-10-14

**Authors:** Huanfen Yang, Zhen Qin, Shengjiao Li, Qingtai Shu, Mingxing Wang, Yiran Zhang, Zeyu Li

**Affiliations:** ^1^ College of Forestry, Southwest Forestry University, Kunming, Yunnan, China; ^2^ Key Laboratory for Forest Resources Conservation and Utilization in the Southwest Mountains of China, Ministry of Education, Kunming, Yunnan, China; ^3^ College of Surveying and Mapping and Information Engineering, West Yunnan University of Applied Sciences, Dali, Yunnan, China; ^4^ College of Soil and Water Conservation, Southwest Forestry University, Kunming, Yunnan, China

**Keywords:** ANUSPLIN interpolation, GEDI, ICESat-2/ATLAS, above-ground carbon storage, machine learning

## Abstract

GEDI and ICESat-2/ATLAS have significant limitations in estimating forest structure parameters. This study aims to enhance the estimation accuracy of above-ground carbon storage (AGC) of *Dendrocalamus giganteus* by applying the ANUSPLIN interpolation technique for spatial expansion. The results indicate that: (1) When the spline degree of ANUSPLIN interpolation was set to 4 for GEDI parameters (cover, pai, sensitivity) and ICESat-2/ATLAS parameters (toc_roughness, h_median_canopy_abs, h_canopy_abs, h_max_canopy_abs), the model’s accuracy was highest. The spline degree 2 for the digital_elevation_model parameter (GEDI) and the asr parameter (ICESat-2/ATLAS) yielded optimal results. (2) The interpolation accuracy and performance of ANUSPLIN outperformed that of co-kriging (CK). (3) The Extreme Gradient Boosting (XGBoost) model (Coefficient of Determination, R^2^ = 0.93; Root Mean Square Error, RMSE = 5.89 Mg/ha; Overall Estimation Accuracy, P = 85.84%; relative RMSE, rRMSE = 14.16%) outperformed the Light Gradient Boosting Machine (LightGBM) (R^2^ = 0.52, RMSE = 14.61 Mg/ha, P = 64.84%, rRMSE = 35.16%) and Random Forest Regression (RFR) (R^2^ = 0.90, RMSE = 8.23 Mg/ha, P = 79.79%, rRMSE = 20.21%), achieving relative improvements of 78.85%, 59.67%, 32.36%, and 59.71% over LightGBM, and 3.33%, 28.41%, 7.58%, and 29.94% over RFR, respectively. This study demonstrates the feasibility of using ANUSPLIN interpolation for satellite LiDAR data from GEDI and ICESat-2/ATLAS. The approach offers a new perspective on spatial interpolation of satellite LiDAR data at a regional scale, providing a valuable reference for cost-effective, high-precision estimation of forest structural parameters.

## 1 Introduction

Above-ground carbon storage (AGC) is one of the most fundamental quantitative characteristics of forest ecosystems, reflecting the complex relationships between material cycling, energy flow, and the interaction between plants and the environment (Sun and Liu, 2019). Bamboo forests, recognized as the “second-largest forest in the world,” are important components of forest ecosystems. As perennial grasses from the subfamily Bambusoideae, their carbon storage accounts for approximately 0.94% of global forest carbon storage (Zhang et al., 2024b). Known as the “Kingdom of Bamboo” (Li et al., 2015; Zhang et al., 2024b), China holds significant bamboo forest resources, with carbon storage of 2.10 × 10^8^ Mg in 2021, accounting for 1.96% of total forest carbon storage. The bamboo forest area spans 7.5627 × 10^6^ ha, representing 3.31% of the national forest area. Compared to the results of the 9th National Forest Resources Survey, the bamboo forest area increased by 17.95% (Feng and Li, 2023). Moreover, studies indicate that while global forest areas have been steadily decreasing, bamboo forest areas have been expanding (Li et al., 2006), suggesting the importance of accurately estimating the AGC of bamboo forests at a regional scale. Such estimates are crucial for understanding global climate change mechanisms, formulating carbon emission policies, and mitigating global warming.

Traditional methods for estimating forest AGC primarily rely on field surveys, which require extensive time and effort, particularly in complex forest structures and regions with variable environmental conditions. These methods struggle to meet the demand for rapid and large-scale forest AGC distribution data (Shu et al., 2022). The advent and development of remote sensing technologies have provided a fast and efficient means of monitoring forest carbon storage (Liu et al., 2024). Current remote sensing studies of bamboo forest biomass and carbon storage mostly use passive optical remote sensing data, but bamboo forests are characterized by high growth density and interwoven branches and stems, making it challenging to estimate AGC storage accurately using optical remote sensing. Research has shown that the estimation accuracy of bamboo biomass using traditional optical remote sensing techniques is relatively low. For instance, Du et al. (2010) estimated *Phyllostachys* edulis above-ground biomass (AGB) based on Landsat TM data, finding a maximum correlation coefficient of 0.48 between AGB and vegetation indices. Chen et al. (2018) used Sentinel-2 data and Random Forest Regression to estimate AGB in bamboo forests of Zhejiang Province, with an R^2^ of 0.46. Yang et al. (2024b) demonstrated that airborne Light Detection and Ranging (LiDAR), based on active remote sensing technology, can improve estimation accuracy, achieving an R^2^ of 0.64. Zhang et al. (2024a) integrated unmanned aerial vehicles (UAV) LiDAR with Sentinel-2 data, achieving an R^2^ of 0.89, though airborne LiDAR is expensive and not suitable for large-scale bamboo forest carbon storage estimation. Yang et al. (2024a) used satellite LiDAR data from Global Ecosystem Dynamics Investigation (GEDI) and Ice, Cloud, and land Elevation Satellite-2/Advanced Terrain Laser Altimeter System (ICESat-2/ATLAS) to estimate bamboo forest AGC at a regional scale, achieving low-cost, high-precision inversion. However, satellite LiDAR provides discrete sampling, which cannot offer full coverage of data, necessitating the use of extrapolation methods. In this context, spatial interpolation, which is an effective way to obtain continuous spatial distribution information, has been widely applied in satellite LiDAR data processing (Yu et al., 2023).

Currently, geostatistical methods such as co-kriging (CK) (Yu et al., 2023) and sequential Gaussian simulation (Luo et al., 2024) have been widely used for spatial interpolation of satellite LiDAR data. However, the use of the ANUSPLIN method for spatial interpolation of satellite LiDAR data is relatively scarce. The effectiveness of interpolation methods is influenced by the study area, and there is no unified standard for selecting an interpolation method. Even the same interpolation technique applied to different regions may yield varying results. Numerous factors affect interpolation accuracy, and selecting the most suitable interpolation method for a given dataset remains a key challenge in current research (Pan, 2021).

ANUSPLIN, based on thin-plate spline theory, was originally developed for interpolating meteorological data (Xu and Hutchinson, 2013). It can incorporate elevation data as a covariate in spatial interpolation. Unlike geostatistical interpolation methods, which require prior calibration of semivariogram parameters, ANUSPLIN is simpler as it does not require such calibration and provides higher interpolation accuracy (Guo et al., 2020). Comparative studies in temperature and precipitation research have shown that ANUSPLIN outperforms other interpolation methods such as ordinary kriging and inverse distance weighting, especially when elevation is used as a covariate in areas with complex terrain (Liu et al., 2012; Peng et al., 2024).

This study focuses on Xinping County in Yunnan Province, where *Dendrocalamus giganteus* is widely distributed, and uses GEDI and ICESat-2/ATLAS satellite LiDAR data as the primary information sources, with elevation data as an auxiliary input. The ANUSPLIN software was employed to perform spatial interpolation of GEDI and ICESat-2/ATLAS parameters, enabling spatial extrapolation. The results were compared with those of CK interpolation in terms of accuracy and performance. Additionally, machine learning models, including Light Gradient Boosting Machine (LightGBM), Random Forest Regression (RFR), and Extreme Gradient Boosting (XGBoost), were used to develop the optimal AGC estimation model for *Dendrocalamus giganteus*. The study evaluated the effectiveness and feasibility of using ANUSPLIN interpolation for GEDI and ICESat-2/ATLAS data, offering valuable insights into the sustainable development of bamboo forests in China.

The specific research questions of this study are: 1) Can ANUSPLIN interpolation significantly improve the spatial extrapolation of spaceborne LiDAR parameters? 2) Are there significant differences in accuracy and visualization performance between ANUSPLIN interpolation and CK? 3) After data expansion using ANUSPLIN interpolation, can machine learning models achieve higher prediction accuracy of *Dendrocalamus giganteus* AGC?

Based on these research questions, we propose the following hypotheses: 1) ANUSPLIN interpolation performs better than CK. 2) The input parameters after ANUSPLIN interpolation can significantly improve the accuracy of AGC models. 3) The XGBoost model outperforms the LightGBM and RFR models in AGC prediction.

The objectives of this study are to propose and validate the applicability of ANUSPLIN interpolation for spaceborne LiDAR data and to evaluate its potential for regional-scale estimation of *Dendrocalamus giganteus* AGC.

## 2 Materials and methods

### 2.1 Study area

**Figure 1:**
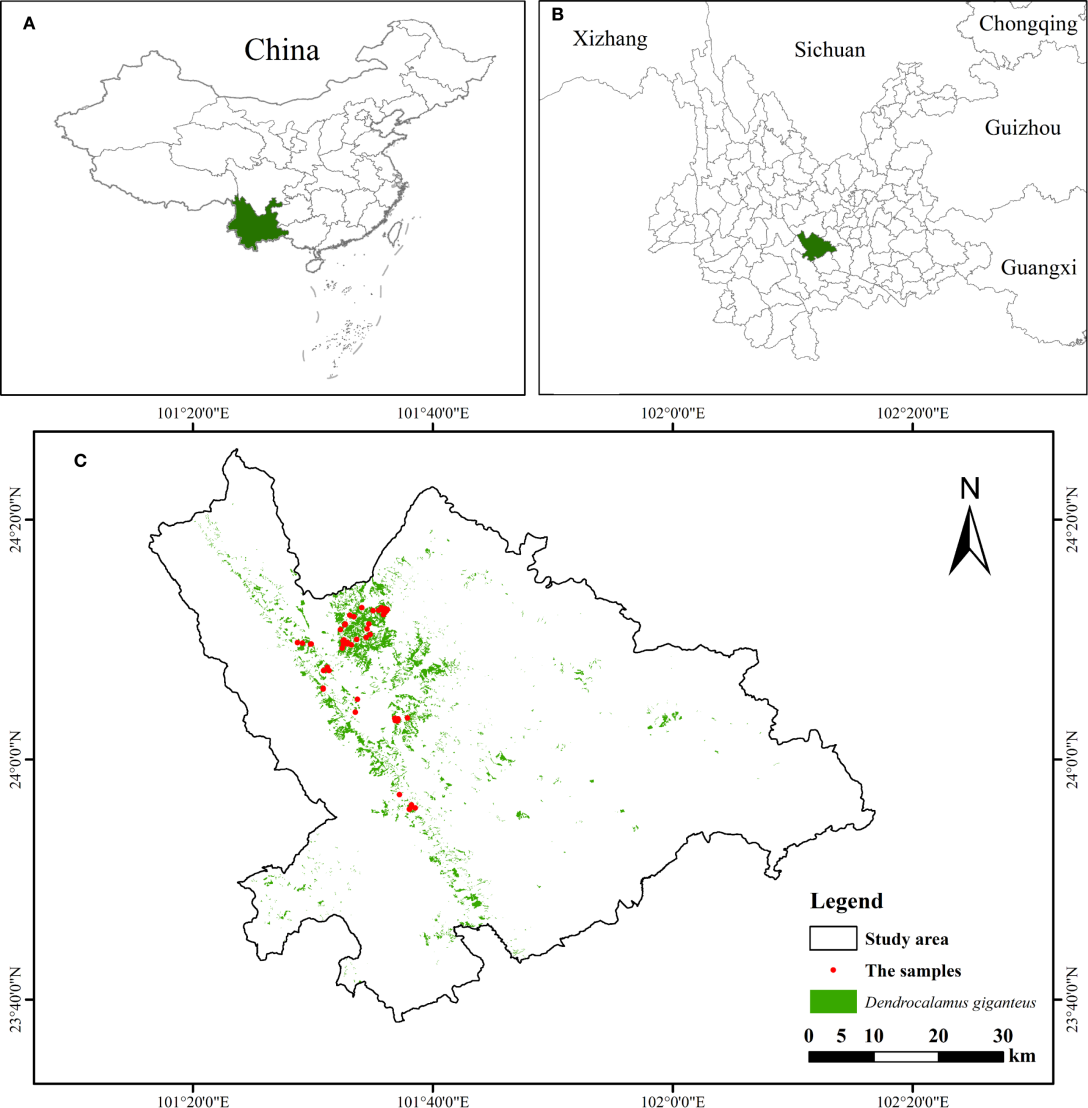
Geographic location map: **(A)** Location of Yunnan Province in China; **(B)** Location of Xinping County in Yunnan Province; **(C)** Xinping.

Xinping County (Qin et al., 2025) (**Figure 1**) is located on the eastern slope of the central Ailao Mountains in southwestern China, with terrain that is higher in the northwest and lower in the southeast. The county lies in a transitional zone with complex topography and fertile soils. Elevation ranges from low valleys to middle mountains, creating diverse habitats. The climate is subtropical to temperate, with a mean annual temperature of about 18 °C, mean annual precipitation of 869 mm, annual sunshine of 2839 h, and a frost-free period of approximately 316 days. Forest land covers 23520.1 ha, accounting for 55.8% of the county’s area, with a forest coverage rate of 70.99%. Vegetation resources are highly diverse, comprising 1402 species of higher plants from 219 families and 762 genera. Bamboo forests occupy 2417.75 ha across 12 townships (about 10% of the forest area), with more than 20 genera represented; among them, *Dendrocalamus giganteus* is dominant, widely distributed between 450 and 1800 m elevation. With the implementation of national ecological and land-use policies, the bamboo industry in Xinping has expanded rapidly and become an important driver of local economic development. Against this background, estimating the carbon storage and biomass of *Dendrocalamus giganteus* provides essential data for forestry production and land-use planning, while supporting sustainable development and China’s goals of achieving carbon peak and carbon neutrality.

### 2.2 Measurement of *Dendrocalamus giganteus* AGC

In Xinping County, Yuxi City, mosquitoes begin to appear in March–April as temperatures gradually rise. From May to November, mosquitoes are more active and abundant, whereas from December to February of the following year, low winter temperatures reduce mosquito activity, and the weather is generally clear. Therefore, the field survey was conducted in January 2024. During the survey, following the principles of representativeness and accessibility, 80 *Dendrocalamus giganteus* plots were preselected based on sub-compartment attribute data to cover different elevations and slopes. Upon arrival in the study area, circular sample plots were established within each preselected sub-compartment according to plant density, growth status, and health, ensuring that each plot included *Dendrocalamus giganteus* of different age classes. Based on the principle of sufficient representativeness and the requirement for a large sample size (approximately 30 plots are considered small, while around 50 plots are regarded as large) (Shu et al., 2022), a total of 51 circular sample plots (radius = 12.5 m, area = 490.63 m²) were established (**Figure 1**).

The center coordinates of each plot were determined using the Qianxun StarMatrix SR3 (Pro version), and data were collected while ensuring the device was in fixed solution mode. In each plot, the diameter at breast height (DBH), geographic position, and plant count were systematically recorded. The estimation of AGC followed three sequential steps: 1) determining the AGB of a representative mean tree; 2) converting the individual AGB to AGC using a carbon content coefficient; 3) calculating plot-level AGC by scaling the individual AGC to the total number of plants within the plot. The corresponding formulas are shown in Equations 1–4:

The AGB model of *Dendrocalamus giganteus* was as follows (Wang et al., 2021):

Bamboo stalk biomass.


(1) $$\begin{array}{c} w=0.145DBH^{2.4197} \end{array}$$

Bamboo biomass.


(2) $$\begin{array}{c} w=0.0224DBH^{2.5286} \end{array}$$

Bamboo leaf biomass.


(3) $$\begin{array}{c} w=0.0196DBH^{1.917} \end{array}$$

The AGC calculation formula of Dendrocalamus giganteus was as follows:


(4) $$\begin{array}{c} C_{t}=W\times f_{c} \end{array}$$

where $C_{t}$ is *Dendrocalamus giganteus* AGC, $f_{c}$ is the carbon coefficient, and the carbon coefficient is 0.45 for bamboo rod, 0.45 for bamboo branch, and 0.43 for bamboo leaf (Teng, 2017).

In these 51 plots, the minimum, maximum, mean, and standard deviation of *Dendrocalamus giganteus* AGC were 4.08 Mg/ha, 101.78 Mg/ha, 41.63 Mg/ha, and 20.55 Mg/ha, respectively, with the calculation procedure following Yang et al. (2024a).

### 2.3 Full waveform data

The GEDI is the first full-waveform spaceborne LiDAR system aboard the International Space Station. It is an active remote sensing technology capable of collecting large-scale data. Using short-wavelength laser pulses, GEDI penetrates the forest canopy to obtain precise three-dimensional forest structure information (Dubayah et al., 2020). Its sampling range extends from 51.6°N to 51.6°S, enabling the inversion of forest structure parameters in all regions except high-latitude areas (Wang et al., 2024). This study utilized the GEDI L2B data product, which is available for download from the official website (https://www.earthdata.nasa.gov). The data used in this study covered the study area from January 2022 to February 2023 and included all accessible swath data. The data is stored in HDF5 format with a spatial resolution of 25 m and consists of discrete laser footprints.

**Figure 2:**
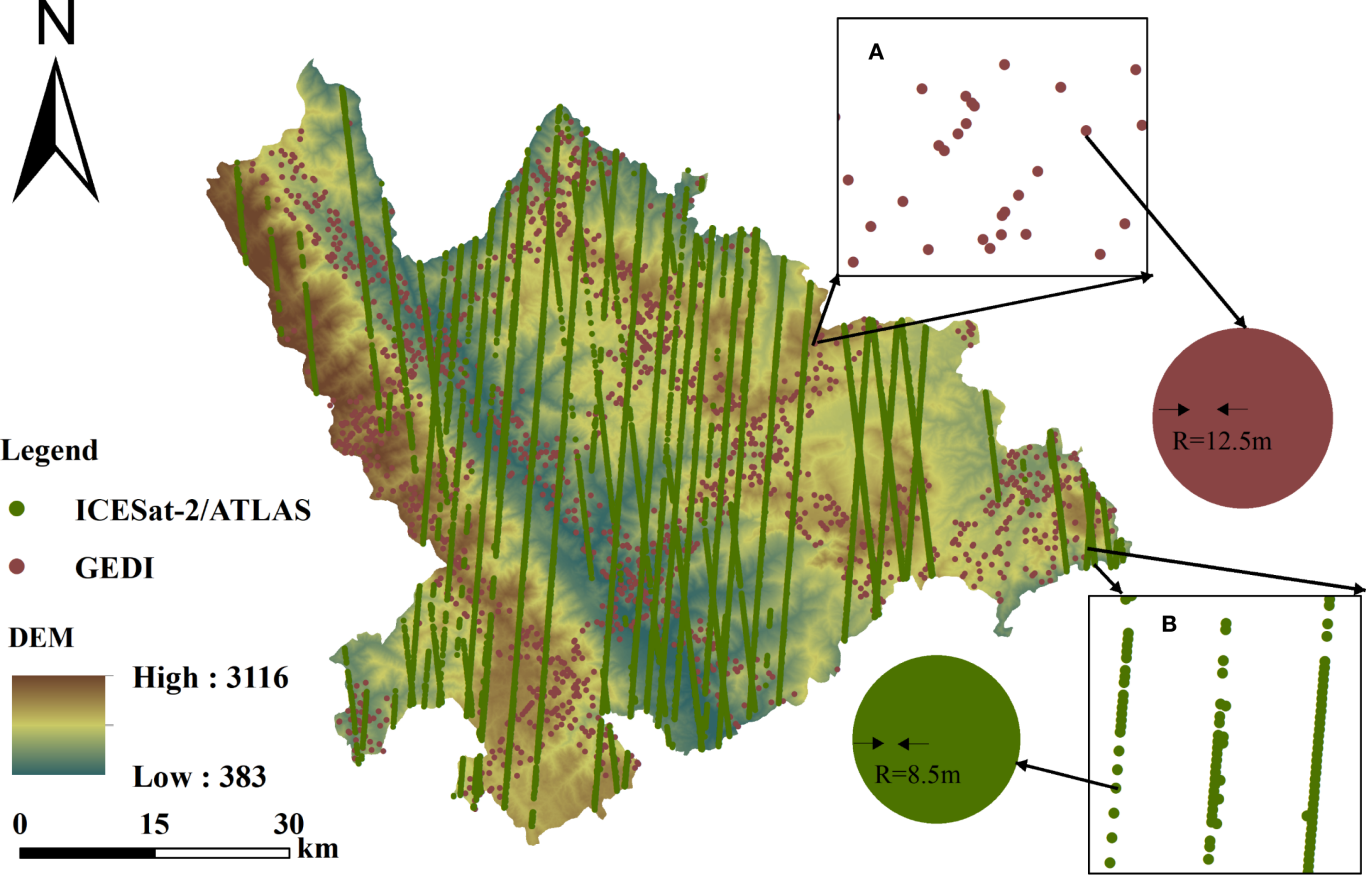
Information source map: **(A)** Localized magnification of GEDI light spots, **(B)** Localized magnification of ICESat-2/ATLAS light spots.

There were a total of 57,217 laser footprint samples in the study area. To ensure uniform and random distribution, systematic sampling was conducted on these footprints (sampling interval: 24). As a result, 2,384 GEDI laser footprint samples were selected as preset samples for spatial analysis (**Figure 2**).

### 2.4 Photon counting

ICESat-2 data products are currently classified into four levels (ATL00-ATL22), with 21 different products. This study primarily uses ATL03 and ATL08 data products. The ATL08 product is derived from ATL03 data using the DDBSCAN (Density-Based Spatial Clustering of Applications with Noise) and KNNB (K-Nearest Neighbor-based) local statistical algorithms, followed by an improved PTD (Progressive Triangulated Irregular Network Densification) classification method. It provides various parameters related to vegetation canopy and terrain (Qin et al., 2024). A comprehensive description of the ICESat-2/ATLAS data products can be accessed on the official website (https://www.earthdata.nasa.gov). This study obtained all available ATL03 and ATL08 data products covering the study area from January 2022 to August 2023.

In the study area, a total of 21,080 laser footprint samples were collected. To ensure uniform and random distribution, systematic sampling was performed on these footprints (sampling interval: 9). As a result, 2,342 ICESat-2/ATLAS laser footprint samples were selected as preset samples for spatial analysis (**Figure 2**).

### 2.5 Auxiliary data

The digital elevation model (DEM) used in this study is the ALOS PALSAR Radiometrically Terrain Corrected (RTC) 12.5 m DEM, obtained from the Alaska Satellite Facility Distributed Active Archive Center (ASF DAAC) via NASA Earthdata Search (https://search.earthdata.nasa.gov/search) (**Figure 2**). The DEM data were resampled to a spatial resolution of 25 m using the Resample tool in ArcMap 10.5 with the bilinear method, in order to match the footprint and plot area.

## 3 Research methods

**Figure 3:**
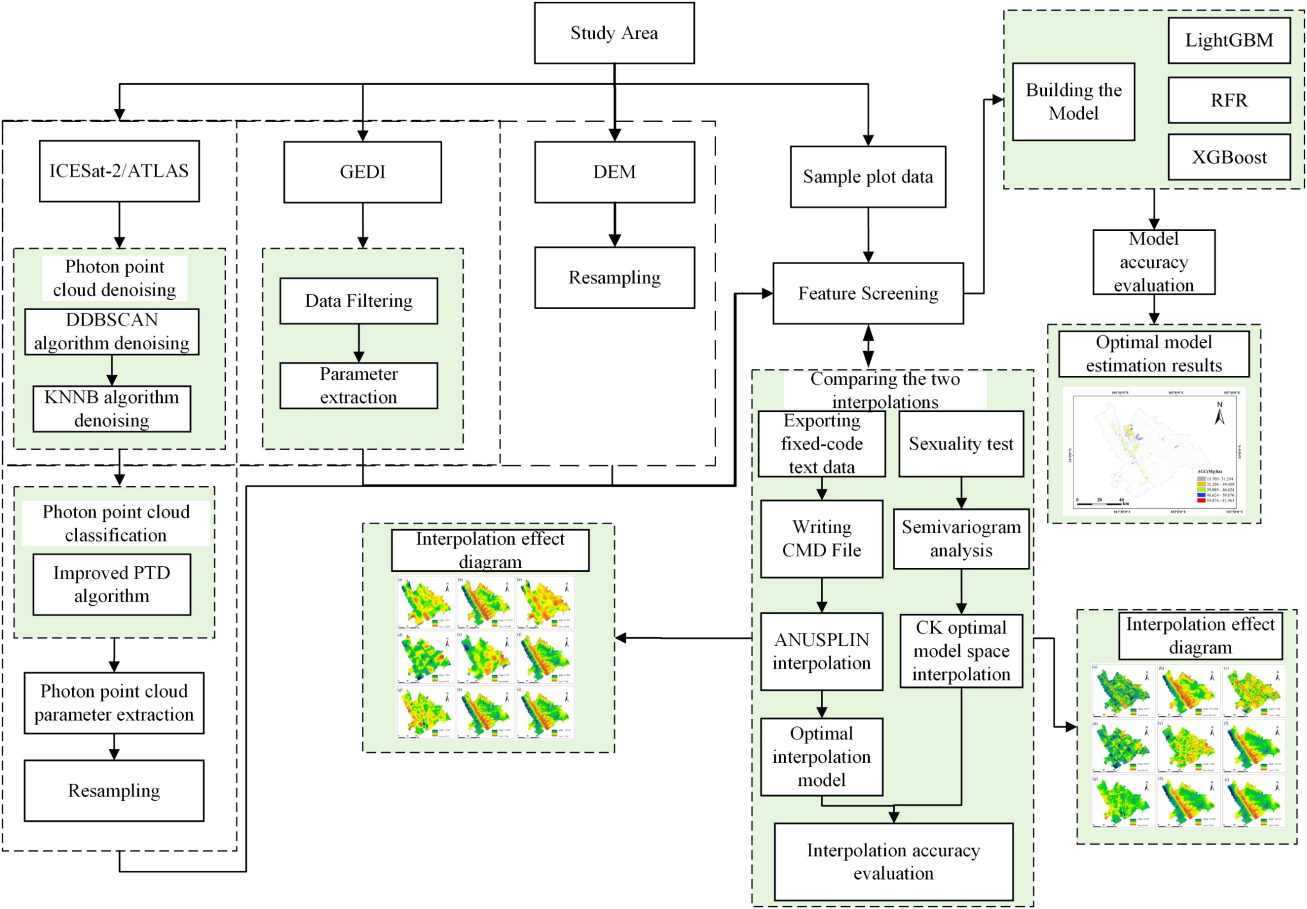
Technical flow chart.

This study employed ANUSPLIN software to interpolate spaceborne LiDAR GEDI and ICESat-2/ATLAS footprint data and compared its performance with CK. A remote sensing estimation model was then constructed to estimate the AGC of *Dendrocalamus giganteus* based on the optimal model (**Figure 3**).

### 3.1 Generation of LiDAR data surface

#### 3.1.1 ANUSPLIN interpolation method

ANUSPLIN is based on the interpolation theory of ordinary thin-plate splines and local thin-plate splines, initially developed by Australian scholars such as Hutchinson. The principle of ANUSPLIN is to use smooth spline functions to interpolate multivariate data, ensuring both the smoothness and accuracy of the interpolation surface (Peng et al., 2024). Local thin-plate smoothing spline interpolation, as an advancement over the traditional thin-plate smoothing spline, not only permits the inclusion of independent variables but also enables the incorporation of covariates such as elevation. The relevant calculations for the ANUSPLIN interpolation method are shown in Equations 5–12.


(5) $$\begin{array}{c} z_{i}=f\left(x_{i}\right)+b^{T}y_{i}+e_{i},\left(i=1,\ldots ,N\right) \end{array}$$

Note: $z_{i}$ is the value of the dependent variable at the i -th point in space, $x_{i}$ is the d-dimensional spline independent variable, the function $f\left(x_{i}\right)$ is a smooth function that estimates $x_{i}$ , $y_{i}$ is the p-dimensional independent covariate, its coefficient is b, and $e_{i}$ is the random error of the independent variable with a mean equal to zero.

The function f and the coefficient b are calculated by least squares estimation:


(6) $$\begin{array}{c} \sum_{i=1}^{N}{\left[\frac{Z_{i}-f\left(x_{i}\right)-b^{T}y_{i}}{w_{i}}\right]}^{2}+pJ_{m}\left(f\right) \end{array}$$

Note: $w_{i}$ is the known local relative coefficient of variation of the weight; $J_{m}\left(f\right)$ is the roughness measure function of function $f\left(x_{i}\right)$ , defined as the partial derivative of f (called spline degree, also called roughness degree); p is a positive smoothness parameter that balances data fidelity and surface roughness. In AUNSPLIN, it is usually determined by minimizing generalized cross-validation GCV and minimizing maximum likelihood GML (Cuervo-Robayo et al., 2014).

The fitted function value vector $\hat{Z}$ is expressed as:


(7) $$\begin{array}{c} \begin{array}{c} \hat{Z}=Az \end{array} \end{array}$$

In Equation 7, A is the influence matrix of size N×N. The degrees of freedom for the fitted spline, based on linear regression principles, are given by:


(8) $$\begin{array}{c} SIGNAL=trace\left(A\right) \end{array}$$

The degrees of freedom for the weighted residual sum of squares is:


(9) $$\begin{array}{c} ERROR=trace\left(I-A\right)=N-trace\left(A\right) \end{array}$$

The weighted mean squared residual is:


(10) $$\begin{array}{c} MSR=\frac{∥W^{-1}\left(I-A\right)z{∥}^{2}}{N} \end{array}$$

In Equation 10, W is a diagonal matrix:


(11) $$\begin{array}{c} W=diag\left(w_{1},\cdots ,w_{N}\right) \end{array}$$

The total degrees of freedom on each surface and the error degrees of freedom sum to N (the total number of spot points). The GCV calculates the value of each smoothing parameter p by leaving out one data point at a time and fitting the surface using the remaining points under a fixed smoothing parameter, then calculating the weighted sum of squares of the residuals between the observed and estimated values. This is the GCV (Hutchinson and Xu, 2004).


(12) $$\begin{array}{c} GCV=\frac{∥W^{-1}\left(I-A\right)z{∥}^{2}/N}{{\left[tr\left(I-A\right)/N\right]}^{2}} \end{array}$$

The specific steps are as follows:(1) Import the interpolation data (xls file) into SPSS and export the data batch file. (2) Place the data file into the ANUSPLIN plugin folder and modify the spline degree, independent, and covariate parameters in the sp.txt file based on the model. (3) Run run.cmd to perform ANUSPLIN interpolation. (4) Convert the generated “grd” file format, process outliers, and perform other operations to obtain the final result.

ANUSPLIN allows for multiple combinations of independent variables, covariates, and spline degrees, resulting in 18 different models (Zhang et al., 2022a). This study used the optimal spatial interpolation model for satellite lidar interpolation with GEDI and ICE-Sat-2/ATLAS parameters as independent variables and elevation as the covariate, setting the spline degree to 2, 3, and 4.

Based on the raw data and the ANUSPLIN user guide, batch command files for running the SPLINA and LAPGRD program modules of ANUSPLIN were written. The SPLINA program module was responsible for generating the coefficients of the surface fitting results and the error statistics file. The LAPGRD program module used the surface coefficient file generated by the SPLINA program module to obtain the interpolation surface. ANUSPLIN provided a range of statistical parameters in the log file, which were used to diagnose error sources and evaluate the interpolation quality. These included the effective number of fitting surface parameters, signal degrees of freedom (Signal), residual degrees of freedom (Error), and signal-to-noise ratio (SNR) (Zhang and Luo, 2011).

Criteria for selecting the best model (Li et al., 2019): Based on generalized cross-validation or the maximum likelihood method, the SNR should have been minimized. The Signal should have been less than half of the number of observation sites, and no “*” symbol should have appeared in the error file generated by SPLINA. The Signal represented the complexity of the fitting surface; if it was greater than half of the observation stations, it indicated significant data errors or the presence of data unsuitable for the surface model. In such cases, the fitting process failed to find the optimal smoothing parameter, and these issues were marked with a “*” symbol in the error statistics file.

In this study, the Generalized Cross Validation (GCV) method provided by ANUSPLIN was used to validate the interpolation results. The evaluation criteria were as follows: the closer the GCV, Mean Squared Residual (MSR), Variance (VAR), Spline Root Mean Squared Error (RTGCV), Spline Residual Root Mean Squared Error (RTMSR), and Spline Variance (RTVAR) were to 0, and the closer the RTGCV value was to the GCV value, RTMSR to MSR, and RTVAR to VAR, the higher the fitting accuracy (Shen, 2012).

#### 3.1.2 CK interpolation method

CK is an extension of ordinary Kriging. Unlike ordinary Kriging, which uses only the spatial autocorrelation of the prediction points, CK utilizes the relationships between the main variable and multiple covariates. The expression can be found in reference (Yu et al., 2023).

### 3.2 Accuracy validation of interpolation results

This study used cross-validation to evaluate the accuracy of the interpolation methods (Liu et al., 2006). The accuracy of the two interpolation methods is compared and analyzed using four metrics: Mean Error (ME), Mean Squared Error (MSE), Root Mean Square Error (RMSE), and Average Standard Error (ASE), with calculation formulas provided in Xu et al. (2023a). The closer the ME and MSE were to 0, the less biased the predicted values were. When the RMSE is minimized (Tian et al., 2011), and the ASE approaches the RMSE, the accuracy improves.

### 3.3 AGC estimation models and their accuracy assessment

In this study, three machine learning regression models—LightGBM, RFR, and XGBoost—were selected and compared for AGC estimation.

The principle of the LightGBM is detailed in references (Schapire, 1990; Li et al., 2024). In the R 4.3.1 environment, the “LightGBM” package was called via the RStudio interface, and a grid search method was applied to optimize and determine the optimal hyperparameter values. Among these parameters, num_leaves (number of leaves), max_depth (maximum depth of trees), and learning_rate (learning rate) were set to 20, 5, and 0.1, respectively.

For details on the principle of RFR, please refer to references (Fan et al., 2018). RFR can evaluate variable importance using metrics such as %IncMSE and IncNodePurity. %IncMSE measures the increase in prediction error when a variable’s values are permuted in Out-of-Bag (OOB) samples, reflecting its contribution to model accuracy, while IncNodePurity sums the reduction in node impurity attributed to each variable across all trees. In this study, the “randomForest” package in RStudio was used to build the RFR model, with importance = TRUE to obtain variable importance. For regression, %IncMSE was adopted for feature selection due to its reliability. The grid search method is applied to optimize and determine the best hyperparameters, including the number of features used for each node split (mtry) and the number of decision trees (ntree), with optimized values of 9 and 200, respectively.

For details about the principles of XGBoost, please refer to references (Jia et al., 2024). In RStudio, the “XGBoost” package is used to implement this method. A grid search method is applied to optimize and determine the best hyperparameter values, with three key parameters: Nrounds (maximum depth of each tree), max_depth (tree depth), and eta (learning rate or step size shrinkage). The optimized values are set to 45, 3, and 0.1, respectively.

This study uses ten-fold cross-validation and evaluates the fitting performance of the constructed regression models using four accuracy metrics: Coefficient of Determination (R^2^), RMSE, Overall Estimation Accuracy (P), and relative RMSE (rRMSE). The calculation formulas can be found in Reference Qin et al. (2025).

## 4 Results

### 4.1 Feature variable selection

**Figure 4:**
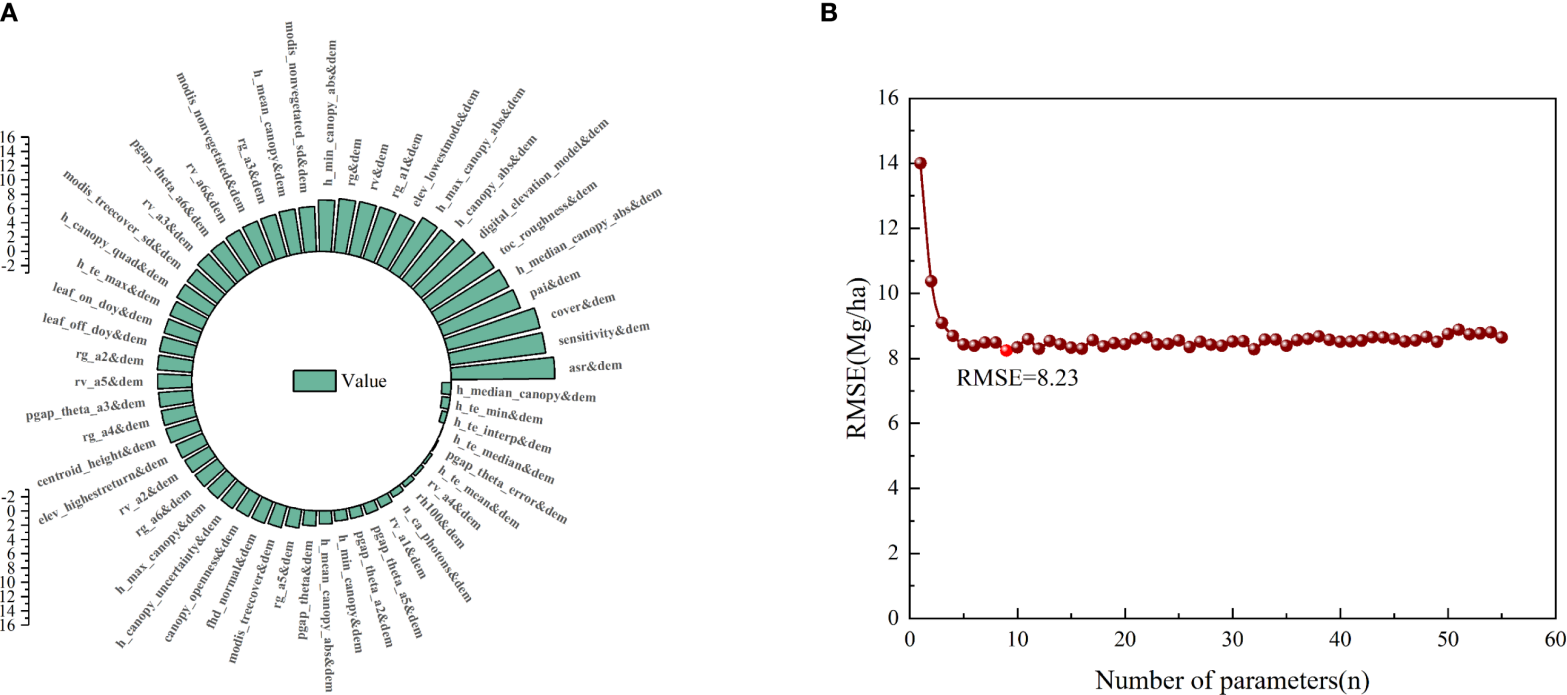
Variable optimization: **(A)** Shows the feature importance ranking, **(B)** Shows the change of RMSE with the number of variables.

Based on the feature importance ranking from the RFR model (**Figure 4A**), the feature variables were sorted in descending order by %IncMSE, where a higher %IncMSE value indicated greater variable importance. By gradually adding variables according to this ranking, the RMSE reached its minimum (8.23 Mg/ha) when the number of variables increased to nine (**Figure 4B**). Therefore, the combination of the top nine variables was selected. Among them, four were GEDI parameters, including cover (13.35%), digital_elevation_model (9.88%), pai (11.54%), and sensitivity (13.48%), while the remaining five were ICESat-2/ATLAS parameters, including toc_roughness (10.80%), h_median_canopy_abs (11.22%), asr (14.53%), h_canopy_abs (8.86%), and h_max_canopy_abs (8.80%). **Table 1** presents the meanings of the final selected variables.

**Table 1:** The optimal variables of GEDI and ICESat-2/ATLAS and their meanings.

Full waveform and photon count data	Variable	Description
GEDI	cover	Total canopy cover is the percentage of ground occupied by the vertical projection of canopy elements.
	digital_elevation_model	WGS84-referenced elevation, calculated from the TandemX 90m product and interpolated at the specified latitude_bin0 and longitude_bin0.
	pai	Total plant area index refers to the combined leaf and stem area per unit ground surface, representing the density of vegetation over a given area.
	sensitivity	The maximum proportion of the canopy that can be effectively sensed, taking into account the signal-to-noise ratio (SNR) of the waveform.
ICESat-2/ATLAS	toc_roughness	Standard deviation of canopy-top photon heights per segment.
	h_median_canopy_abs	Segment-level median of absolute canopy heights (WGS84).
	asr	The proportion of incoming solar radiation that is reflected from the surface, as measured by remote sensing instruments.
	h_canopy_abs	98% of individual canopy heights referenced to WGS84 per segment.
	h_max_canopy_abs	Maximum canopy height above WGS84 per segment.

### 4.2 Generation and comparison of LiDAR data surfaces

#### 4.2.1 ANUSPLIN

As shown in **Table 2**, the three parameters of GEDI, namely cover, pai, and sensitivity, and the four parameters of ICESat-2/ATLAS, including toc_roughness, h_median_canopy_abs, h_canopy_abs, and h_max_canopy_abs, yielded the highest model accuracy when the number of spline functions was set to 4. This accuracy was significantly higher than that obtained with spline numbers of 2 and 3. In contrast, for the GEDI parameter digital_elevation_model and the ICESat-2/ATLAS parameter ASR, the model achieved higher accuracy when the spline number was set to 2 than when it was set to 3 or 4.

**Table 2:** Statistical characteristics of ANUSPLIN interpolation output for GEDI and ICESat-2/ATLAS data.

Full waveform and photon count data	Variable	Spline counts	Error	Signal	SNR	With or without “*”
GEDI	cover	2	2258.5	97.5	0.043	NO
		3	2258.5	97.5	0.043	NO
		4	2266.8	89.2	0.039	NO
	digital_elevation_model	2	2351.4	4.6	0.002	NO
		3	2349	7.0	0.003	YES
		4	2343.1	12.9	0.006	NO
	pai	2	2018.5	337.5	0.167	NO
		3	2106.7	249.3	0.118	NO
		4	2251.4	104.6	0.046	NO
	sensitivity	2	2042.1	313.9	0.154	NO
		3	2092.9	263.1	0.126	NO
		4	2104.8	251.2	0.119	NO
ICESat-2/ATLAS	toc_roughness	2	2301.1	127.9	0.056	NO
		3	2329.0	100.0	0.043	NO
		4	2337.1	91.9	0.039	NO
	h_median_canopy_abs	2	1012.3	1416.7	1.399	NO
		3	1596.5	832.5	0.521	NO
		4	2329.5	99.5	0.043	NO
	asr	2	1831.8	554.2	3.305	NO
		3	2129.1	256.9	8.288	NO
		4	2151.1	234.9	9.158	NO
	h_canopy_abs	2	1033.6	1395.4	1.350	NO
		3	1608.8	820.2	0.510	NO
		4	2333.1	95.9	0.041	NO
	h_max_canopy_abs	2	1035.1	1393.9	1.347	NO
		3	1612.9	816.1	0.510	NO
		4	2333.8	95.2	0.041	NO

From **Table 3**, it can be seen that among the nine variables, except for the digital_elevation_model variable, where the values of the five evaluation indicators (GCV, MSR, VAR, RTGCV, RTMSR, RTVAR) were relatively large, the evaluation indicators for the other eight variables tended to 0. This indicated that ANUSPLIN interpolation was suitable for the spatial expansion of spaceborne LiDAR, and the experimental results were reliable.

**Table 3:** Statistical analysis of generalized cross-validation.

Full waveform and photon count data	Variable	Mean value of the fitted surface metrics			Mean values of fitted surface metrics		
		GCV	MSR	VAR	RTGCV	RTMSR	RTVAR
GEDI	cover	0.09	0.08	0.09	0.30	0.29	0.30
	digital_elevation_model	183.00	183.00	183.00	13.50	13.50	13.50
	pai	3.47	3.16	3.31	1.86	1.78	1.82
	sensitivity	0.00	0.00	0.00	0.02	0.02	0.02
ICESat-2/ATLAS	toc_roughness	0.11	0.10	0.10	0.33	0.32	0.32
	h_median_canopy_abs	0.15	0.140	0.140	0.39	0.38	0.38
	asr	0.00	0.00	0.00	0.07	0.05	0.06
	h_canopy_abs	0.148	0.137	0.142	0.385	0.370	0.377
	h_max_canopy_abs	0.148	0.136	0.142	0.384	0.369	0.377

#### 4.2.2 CK

Select the interpolation model based on the optimal principle of the semivariogram function (Qin et al., 2025), **Table 4** shows the following: For the four GEDI parameters—cover, digital_elevation_model, pai, and sensitivity—the optimal model was the Gaussian model. For the five ICESat-2/ATLAS parameters—toc_roughness, h_median_canopy_abs, asr, h_canopy_abs, and h_max_canopy_abs—the optimal model was the Spherical model. Among the nine parameters, only GEDI’s digital_elevation_model and ICESat-2/ATLAS’s h_median_canopy_abs exhibited block effects in the range of 25% - 75%, indicating moderate spatial autocorrelation. The other seven parameters showed strong spatial autocorrelation.

**Table 4:** Variogram models and comparison assessment of GEDI and ICESat-2/ATLAS factors.

Full waveform and photon count data	Variable	Model	C_0_	C_0_+C	C_0_/C_0_+C	a/m	RSS	R^2^
GEDI	cover	Gaussian	0.1	53.71	0.19	12817.18	715	0.82
		Spherical	0.1	53.52	0.19	16100	777	0.81
		Exponents	0.1	53.87	0.19	18900	1226	0.71
	digital_elevation_model	Gaussian	2000	354800	0.56	14895.64	2.81×10^10^	0.833
		Spherical	1000	353300	0.28	18600	3.09×10^10^	0.833
		Exponents	1000	355500	0.28	21600	5.06×10^10^	0.736
	pai	Gaussian	0.1	293	0.03	13683.2	2.86×10^4^	0.765
		Spherical	0.1	291.6	0.03	17000	3.13×10^4^	0.763
		Exponents	0.1	291.9	0.03	19200	4.64×10^4^	0.659
	sensitivity	Gaussian	0.001	1.216	0.082	16627.69	1.59	0.561
		Spherical	0.001	1.213	0.082	21200	1.63	0.565
		Exponents	0.001	1.203	0.083	22500	2.03	0.472
ICESat-2/ATLAS	toc_roughness	Gaussian	6.67	19.33	34.50	19572.17	78.50	0.726
		Spherical	5.04	19.46	25.90	25200	72.3	0.748
		Exponents	0.01	19.37	0.05	20700	74.4	0.740
	h_median_canopy_abs	Gaussian	103	775.5	13.28	24941.53	40386	0.94
		Spherical	15	776.8	1.93	30400	35443	0.95
		Exponents	1	804.3	0.12	37800	77303	0.90
	asr	Gaussian	-0.08	-2.002	3.99	5022.95	14	0.072
		Spherical	-0.001	-2	0.05	6300	14	0.075
		Exponents	-0.001	-1.983	0.05	5700	14.5	0.048
	h_canopy_abs	Gaussian	102	772	13.21	24941.53	40326	0.945
		Spherical	15	773.2	1.93	30400	35402	0.952
		Exponents	1	800.6	0.12	37800	76975	0.904
	h_max_canopy_abs	Gaussian	102	771.4	13.22	24941.53	40312	0.945
		Spherical	15	772.6	1.94	30400	35386	0.952
		Exponents	1	800	0.12	37800	76905	0.904

#### 4.2.3 Comparison of ANUSPLIN and CK interpolation accuracy

From **Table 5**, it could be observed that, among the nine parameters, ANUSPLIN interpolation showed better accuracy than CK interpolation for all parameters except for digital_elevation_model, where the MSE and ASE were higher for ANUSPLIN than for CK. For the other parameters, ANUSPLIN outperformed CK in terms of accuracy.

**Table 5:** Cross-validation results for ANUSPLIN and CK.

Full waveform and photon count data	Variable	CK				ANUSPLIN			
		ME	RMSE	MSE	ASE	ME	RMSE	MSE	ASE
GEDI	cover	0.000	0.310	0.000	0.323	0.000	0.289	0.000	0.083
	digital_elevation_model	-0.205	108.932	-0.030	91.821	-0.123	13.892	192.975	192.975
	pai	-0.006	2.001	-0.001	1.899	0.000	1.656	0.000	1.743
	sensitivity	0.000	0.0187	0.001	0.0134	0.000	0.016	0.000	0.000
ICESat-2/ATLAS	toc_roughness	-0.023	0.359	-0.005	0.213	0.000	0.321	0.003	0.103
	h_median_canopy_abs	-0.007	0.448	-0.001	0.522	0.000	0.290	0.000	0.138
	asr	0.001	0.079	0.009	0.055	0.000	0.070	0.005	0.005
	h_canopy_abs	0.000	0.447	0.000	0.520	0.000	0.434	0.000	0.508
	h_max_canopy_abs	0.000	0.446	0.000	0.520	0.000	0.290	0.000	0.139

**Figure 5:**
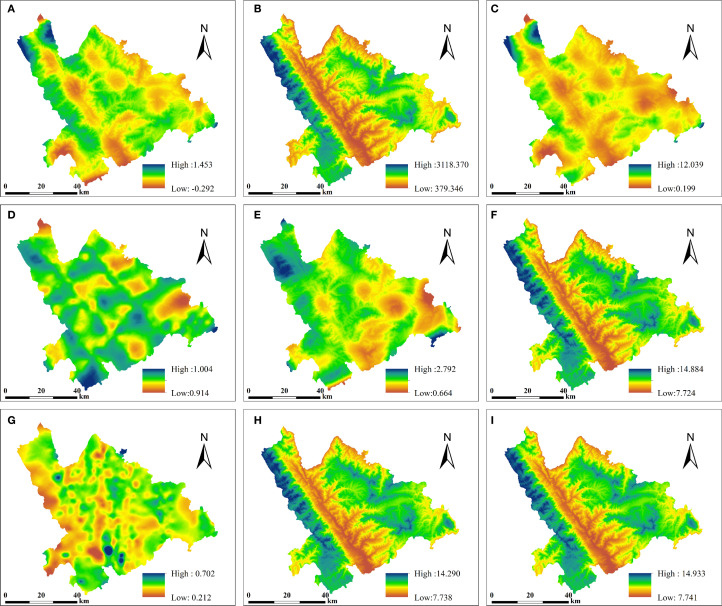
ANUSPLIN interpolation results **(A-D)** GEDI parameters: **(A)** cover, **(B)** digital_elevation_model, **(C)** pai, **(D)** sensitivity. **(E-I)** ICESat-2/ATLAS parameters: **(E)** toc_roughness, **(F)** h_median_canopy_abs, **(G)** asr, **(H)** h_canopy_abs, **(I)** h_max_canopy_abs.

**Figure 6:**
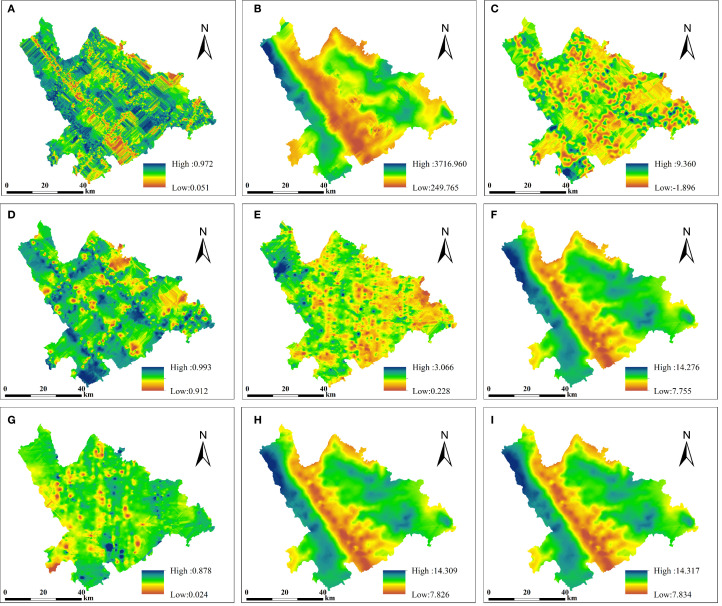
CK interpolation results **(A-D)** GEDI parameters: **(A)** cover, **(B)** digital_elevation_model, **(C)** pai, **(D)** sensitivity; **(E-I)** ICESat-2/ATLAS parameters: **(E)** toc_roughness, **(F)** h_median_canopy_abs, **(G)** asr, **(H)** h_canopy_abs, **(I)** h_max_canopy_abs.

The interpolation results from the two methods are presented (**Figures 5**, **6**). Compared with CK, ANUSPLIN produced smoother surfaces and better preserved local details, reducing the striping effect to some extent. This improvement may be due to the fact that the empirical covariance function only approximates spatial covariance variation, and the fitting process inevitably leads to some loss of local detail. The resulting surface files were converted to TIFF format using ArcGIS 10.5 for visualization (**Figure 5**), where the details of the nine variables were clearly highlighted, displaying distinct “mosaic” patterns. ANUSPLIN interpolation also demonstrated strong natural smoothing, effectively capturing terrain characteristics and clearly delineating mountainous contours in areas of complex topography. In contrast, **Figure 6** shows that panels (A), (D), (E), and (G) exhibit pronounced striping effects, while panels (A–E, G) display mechanical gradients characterized by uneven or bumpy patterns.

### 4.3 Regional-scale model estimation and mapping results

**Figure 7:**
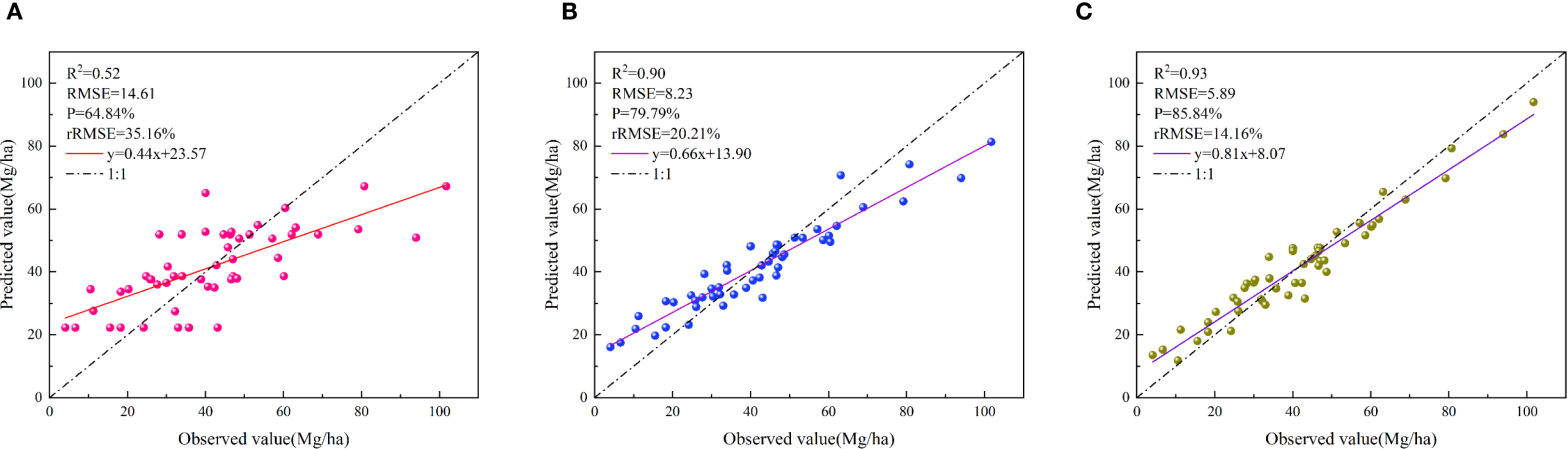
Scatter plots of *Dendrocalamus giganteus* AGC models **(A)** LightGBM, **(B)** RFR, **(C)** XGBoost.

The 9 GEDI and ICESat-2/ATLAS independent variables selected using Random Forest feature importance, together with data from 51 plots, were input into the LightGBM, RFR, and XGBoost models. The evaluation metrics (R^2^, RMSE, P, rRMSE) for the three models varied (**Figure 7**). The XGBoost model (R^2^ = 0.93, RMSE = 5.89 Mg/ha, P = 85.84%, rRMSE = 14.16%) outperformed the LightGBM (R^2^ = 0.52, RMSE = 14.61 Mg/ha, P = 64.84%, rRMSE = 35.16%) and RFR (R^2^ = 0.90, RMSE = 8.23 Mg/ha, P = 79.79%, rRMSE = 20.21%), achieving relative improvements of 78.85%, 59.67%, 32.36%, and 59.71% over LightGBM, and 3.33%, 28.41%, 7.58%, and 29.94% over RFR, respectively.

**Figure 8:**
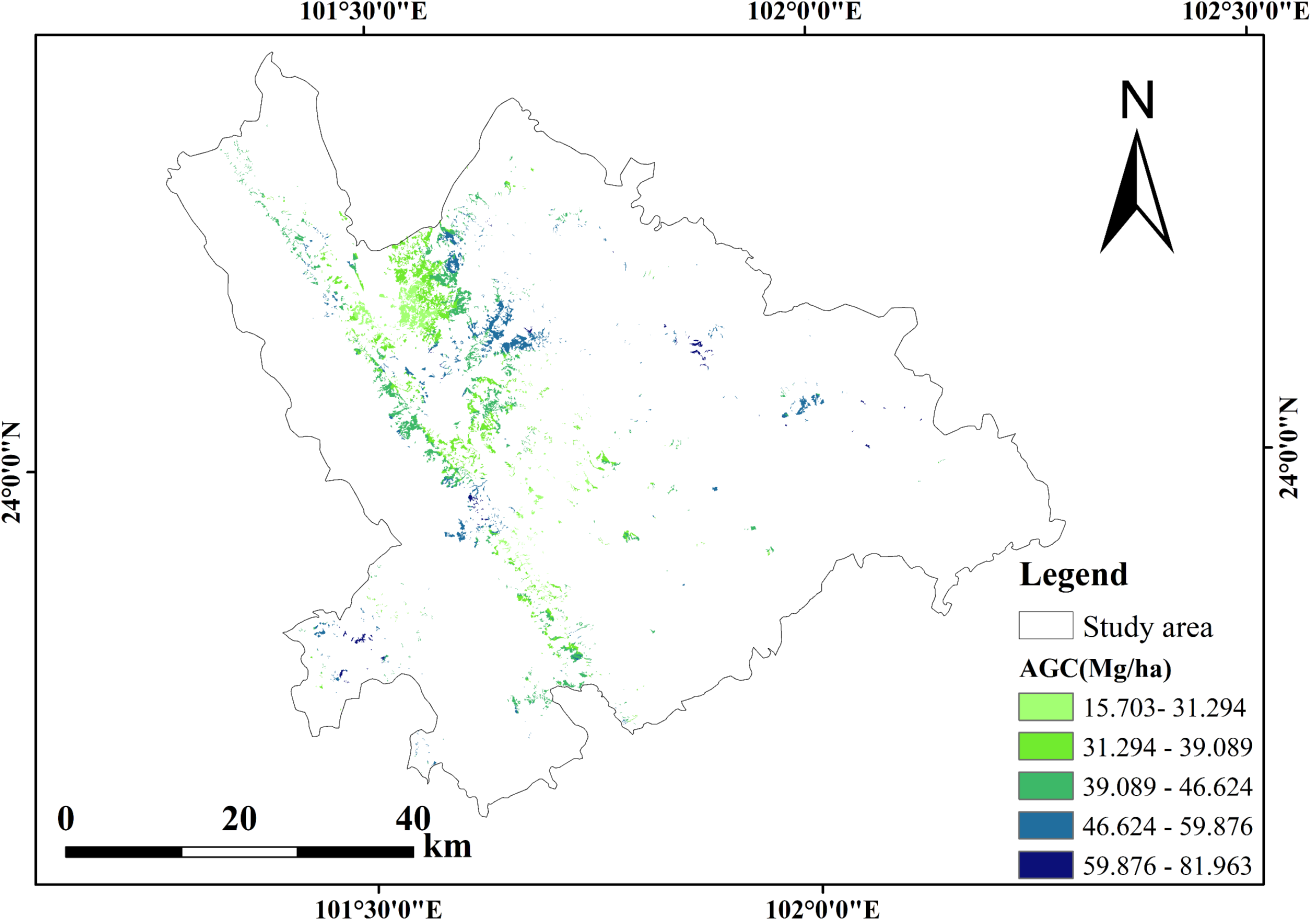
AGC distribution map of *Dendrocalamus giganteus* in Xinping.

Based on the XGBoost model, the AGC distribution of *Dendrocalamus giganteus* in Xinping County was mapped (**Figure 8**). The average AGC for the 51 plots predicted by XGBoost was 41.59 Mg/ha. Across Xinping County, the average AGC was 40.62 Mg/ha, ranging from 15.70 to 81.96 Mg/ha, with a total stock of 1.14 × 10^7^ Mg. Spatially, most *Dendrocalamus giganteus* was concentrated in the northwest of the county, with fewer distributions in other areas. The species was mainly found at elevations between 373 and 1755 m, with only a small proportion occurring above 1755 m, consistent with field observations.

## 5 Discussion

### 5.1 The impact of different sensors on bamboo forest AGC estimation

AGC prediction based on spectral data generally shows lower accuracy than that using LiDAR data. This is likely because multispectral data only provide horizontal information about the canopy structure and cannot capture vertical structural information. However, vertical structural parameters, such as tree height and canopy height, are closely related to the AGC of the forest (Su et al., 2023). For example, Li et al. (2018) estimated the biomass of *Phyllostachys* edulis in Zhejiang Province using MODIS time-series data and a random forest algorithm, with an R^2^ value of 0.53 for accuracy validation. Zhao (2016) used Landsat TM and ALOS PALSAR data for bamboo forest biomass by developing different estimation models based on single and combined data sources. Their study found that bamboo forests have relatively lower biomass saturation compared to other forest types. When the biomass exceeds 75 Mg/ha, remote sensing data tends to saturate, and spectral reflectance no longer changes with biomass, making it difficult to accurately estimate biomass beyond the saturation point. This leads to lower model estimation accuracy (Zhang et al., 2006).

LiDAR, on the other hand, can quickly acquire three-dimensional spatial information, has strong penetration capabilities, and can precisely monitor vertical canopy structure parameters. It offers a significant advantage in forest parameter inversion, biomass, and carbon stock estimation, leading to greatly improved estimation accuracy (Korhonen et al., 2011; Peduzzi et al., 2012). For instance, Cai (2016) developed bamboo biomass estimation models using airborne high-resolution imagery and airborne LiDAR data, and the LiDAR-based model achieved a correlation coefficient of 0.8. However, the high cost of airborne data acquisition limits its applicability to regional-scale studies. In contrast, spaceborne LiDAR offers global coverage and lower acquisition costs, making it a promising data source for future bamboo biomass and carbon stock estimation. Compared with the study by Yang et al. (2024a), which employed CK interpolation, the estimation accuracies of the XGBoost and RFR models were R^2^ = 0.90, RMSE = 7.62 Mg/ha, P = 81.66%, and R^2^ = 0.88, RMSE = 9.06 Mg/ha, P = 78.20%, respectively. In this study, after applying ANUSPLIN interpolation, the accuracies of the XGBoost and RFR models were further improved to R^2^ = 0.93, RMSE = 5.89 Mg/ha, P = 85.84%, and R^2^ = 0.90, RMSE = 8.23 Mg/ha, P = 79.79%, respectively. These results demonstrate that ANUSPLIN interpolation can effectively enhance the spatial extrapolation of spaceborne LiDAR data and improve model prediction accuracy to some extent. This provides more robust technical support for bamboo carbon stock monitoring and regional-scale forest carbon sink assessment, contributing positively to the achievement of carbon peaking and carbon neutrality goals in the forestry sector.

### 5.2 The impact of interpolation methods on the effect of map visualization

Based on the ANUSPLIN interpolation method, space-borne lidar was combined for the first time for spatial expansion. The interpolation accuracy evaluation was consistent with the conclusions obtained from other interpolation accuracy verifications, indicating that the results of the article are reliable. This is one of the innovations of this study. Liu et al. (2021) showed that the ANUSPLIN method has significant advantages in precipitation interpolation, but the choice of DEM resolution can affect interpolation accuracy. Their conclusion was that, for the same region, different DEM resolutions rank as follows: 500 m resolution ≥ 1 km resolution > 2 km resolution. Xiao et al. (2023) found that, under simulations using three different DEM resolutions, temperature interpolation accuracy followed the order of 25 m > 90 m > 1 km. In this study, a 12.5 m DEM was used, and to standardize the resolution, it was resampled to 25 m. This DEM data is beneficial for improving ANUSPLIN interpolation accuracy. Furthermore, the number of splines significantly impacts result accuracy, and ANUSPLIN interpolation is highly dependent on model selection (Xu et al., 2023b).

Through ANUSPLIN interpolation of satellite LiDAR data and selection of the optimal model based on established principles, the results, verified by cross-validation (**Table 5**), show that this method outperforms geostatistical interpolation methods (Yu et al., 2023; Xu et al., 2023a). From a visual perspective, ANUSPLIN results are superior to those of CK, likely due to CK’s use of neighboring known points combined with semivariance analysis to build a statistical model, which is susceptible to statistical patterns and is prone to creating striping effects, especially in areas with many spots and high spatial heterogeneity. ANUSPLIN, on the other hand, fits a curved surface that minimizes curvature using control points (Bookstein, 1989), yielding a smoother overall result. Compared to CK, ANUSPLIN interpolation maintains accuracy, smoothness, and detail in the interpolation surface. It is also more efficient and requires fewer manual parameter adjustments, thus reducing uncertainty in the interpolation process. This conclusion can offer useful insights for LiDAR data interpolation applications. However, this study only considered elevation data as a covariate, while other terrain factors, such as aspect and slope, also significantly affect interpolation results. Future studies will consider incorporating multiple covariates into the ANUSPLIN interpolation method, providing a more meaningful reference for optimizing LiDAR interpolation accuracy. Additionally, this study focused only on *Dendrocalamus giganteus* because it is widely distributed in Xinping County. However, Yunnan Province has diverse vegetation types, so future research should explore the applicability of ANUSPLIN interpolation using LiDAR data for other vegetation types.

### 5.3 The impact of different spot densities of satellite LiDAR on interpolation

In model development, sample size affects the accuracy of the model. Generally, the larger the sample size, the better the model’s reliability, though too large a sample size can waste resources (Shu et al., 2022). Shu et al. (2022) suggested that to reduce human effort and time, optimal sample sizes need to be explored. This issue also applies to satellite LiDAR spot densities: the more spots there are, the longer the interpolation takes, and the more prominent the striping effect becomes. This is because the satellite LiDAR footprint points are evenly distributed along the orbit. Before interpolating spot data, GEDI data undergo quality screening according to a selection criterion, filtering out low-quality spots from the same or adjacent strips to enhance the spatial randomness of the footprint points (Xu et al., 2023a). When extracting ICESat-2/ATLAS parameters, the effective number of photon point clouds after denoising and classification algorithms on the raw ATL03 data reached tens of thousands (Bookstein, 1989). However, even after preprocessing, the remaining spots from both GEDI and ICESat-2/ATLAS still do not meet the spatial randomness requirement. Some researchers have carried out point thinning before interpolation to alleviate the striping effect (Yu et al., 2023), without affecting accuracy. This further suggests that future studies could explore the optimal number of spot points for interpolation. Zhu et al. (2020) discussed the interpolation accuracy of three algorithms (PRISM, CK, and IDW) at different station densities. They concluded that errors increase as the sample size decreases. Regarding interpolation mapping, when the sample size is small, CK interpolation performs relatively better and results in smoother maps. This may be because the distance between the sample and grid points increases, corresponding to the smoother parts of the empirical semivariance function. Therefore, future research could explore the impact of spot density on interpolation, seeking the optimal number of spots to reduce the workload.

### 5.4 The impact of variable selection on model accuracy

Variable selection is a prerequisite for model development. The choice of modeling variables directly influences the predictive performance of the inversion model. By selecting relevant variables, non-contributory ones can be eliminated, reducing data dimensions, simplifying the model, and enhancing both model accuracy and generalization ability (Luo et al., 2022). When estimating forest structural parameters, different variable selection methods lead to variations in model prediction accuracy, depending on the data combinations, thus affecting the estimation results (Zhang et al., 2022b). For instance, Zhou et al. (2024) employed three methods—Pearson correlation, Recursive Feature Elimination (RFE) using RFR, and Support Vector Machines (SVM-RFE)—to select optimal feature variables for remote sensing models. Their study showed significant differences in the accuracy of remote sensing models constructed with different feature selection methods. Models built with parameters selected by SVM-RFE and RFR demonstrated better precision. Due to the complex relationship between the AGC of *Dendrocalamus giganteus* and satellite LiDAR parameters, variable selection becomes crucial. This study employed the Random Forest feature importance method for variable selection, ensuring that more explanatory and influential variables were prioritized (Zhou et al., 2024). Through this feature importance ranking, variables were progressively added to the model. When the number of variables reached nine, the RMSE was minimized (RMSE = 8.23 Mg/ha). Future research can explore additional methods, such as SVM-RFE, Boruta, and KNN-FIFS, for feature selection, aiming to identify even more optimal parameters for improving the predictive accuracy of satellite LiDAR estimation models.

### 5.5 Prospects for future research

A methodological highlight of this study is the first application of the ANUSPLIN interpolation method for spatial extrapolation of spaceborne LiDAR data, demonstrating its superiority over the conventional geostatistical CK method. In addition, an XGBoost-based model was developed to estimate AGC in bamboo forests with higher accuracy. The results have important implications for bamboo carbon stock monitoring, forest resource surveys, and regional carbon sink assessment. Nevertheless, this study has some limitations, such as the incomplete consideration of covariates, the focus on a single species (*Dendrocalamus giganteus*), and a limited number of sample plots. Future research could incorporate additional topographic and environmental factors, increase the number of sample plots and LiDAR footprints, and validate the method across different forest types and larger regions, thereby providing more reliable technical support for forest carbon stock estimation and carbon neutrality targets.

## 6 Conclusion

This study systematically explored spatial interpolation methods using GEDI and ICESat-2/ATLAS data and evaluated their performance in estimating the AGC of *Dendrocalamus giganteus*. The results showed that ANUSPLIN outperformed CK interpolation. When nine key variables were selected based on random forest feature importance (GEDI: cover, digital_elevation_model, pai, sensitivity; ICESat-2/ATLAS: toc_roughness, h_median_canopy_abs, asr, h_canopy_abs, h_max_canopy_abs), the RMSE was reduced to 8.23 Mg/ha. Among the machine learning models, XGBoost performed best with these variables (R^2^ = 0.93, RMSE = 5.89 Mg/ha, P = 85.84%, rRMSE = 14.16%), predicting an average AGC of 40.62 Mg/ha for *Dendrocalamus giganteus* and a total AGC of 1.14 × 10^7^ Mg in Xinping County. This study demonstrates that the framework integrating ANUSPLIN with spaceborne LiDAR has strong potential for large-scale forest carbon stock monitoring and provides valuable references for climate change research.

## Data Availability

The raw data supporting the conclusions of this article will be made available by the authors, without undue reservation.
